# Medicine in the Gutter

**Published:** 1901-06

**Authors:** 


### *EDITORIAL NOTES AND COMMENTS.*

---

The Business office of THE JOURNAL-RECORD is No. 64 Marietta Street.

The Editorial office is 318-319-320 The Prudential.

Address all Business communications to Dr. M. B. Hutchins, Mgr.

Make remittances payable to THE ATLANTA JOURNAL-RECORD OF MEDICINE.

On matters pertaining to the Editorial and Original Communications address Dr. Bernard Wolff, Atlanta.

Reprints of original articles will be furnished at cost price. Requests for the same should always be made *on the manuscript*.

We will present, post-paid, on request, to each contributor of an original article, twenty (20) marked copies of THE JOURNAL-RECORD containing such article.

---

### "MEDICINE IN THE GUTTER."

THE *Australasian Medical Gazette* for April 20 contains an editorial entitled "Medicine in the Gutter." The phrase is applied to the habit of medical men of endorsing proprietary articles, and to the means adopted by drug concerns in exploiting their wares. It states that a large proportion of this literature comes to us from our cousins over the Pacific," and continuing in this strain, it infers that this special order of vice is intrinsically American. It cannot be denied that the "testimonial habit," borrowed, probably, from the methods of the charlatan, is unfortunately very prevalent, and adds nothing to the dignity of the profession; but that these flowers of literature are cultivated solely on the soil of the great Republic is easily demonstrated as untrue. A perusal of the circulars sent out by the proprietors of protected combinations will show that not a few of those who lend their aid to dragging medicine "in the gutter" carry after their names those alphabetical appendages bestowed only by the medical schools of "the right little, tight little island," to say nothing of endorsements from the

London *Lancet* and other presumably respectable British medical publications. It may be noted that the *Gazette* itself fills the larger part of its advertising space with advertisements of American firms whose methods of getting the goods before the profession it so deplores, and thus it becomes in a measure *particeps criminis.* If the Americans are offenders in regard to furnishing testimonial letters how much greater is the offense committed by the Germans! The American testimonial is generally the bare narrative of a clinical case, the German is more like a chapter in a text-book with a wealth of original work worthy of a better cause, and the writer is almost invariably a "privat-docent," a "saintätsrath" or he possesses some manner of "tone-lending" distinction.

Criticism is welcomed when it is intelligent and disinterested, otherwise, one may rub along very well without it.

Perhaps, without attempting justification of a palpable, but by no means exclusive fault, the suggestion might be ventured that if the dwellers by "the long wash of Australasian seas" would put a little more ginger in their business methods their island might be known for something else than boomerangs and kangaroos. There has been, however, one book produced by the Australian medical contingent which is destined to stick in the memory on account of the truly wonderful name of its author—Balls-Headley.

